# A Comparative Study on the Wettability of Unstructured and Structured LiFePO_4_ with Nanosecond Pulsed Fiber Laser

**DOI:** 10.3390/mi12050582

**Published:** 2021-05-20

**Authors:** Mulugeta Gebrekiros Berhe, Dongkyoung Lee

**Affiliations:** 1 Department of Future Convergence Engineering, Kongju National University, Cheonan 31080, Korea; mule1041@gmail.com; 2 Department of Mechanical and Automotive Engineering, Kongju National University, Cheonan 31080, Korea

**Keywords:** wettability, electrodes, laser structuring, spread area, electrolyte, wetting time

## Abstract

The wettability of electrodes increases the power and energy densities of the cells of lithium-ion batteries, which is vital to improving their electrochemical performance. Numerous studies in the past have attempted to explain the effect of electrolyte and calendering on wettability. In this work, the wettability behavior of structured and unstructured LiFePO_4_ electrodes was studied. Firstly, the wettability morphology of the structured electrode was analyzed, and the electrode geometry was quantified in terms of ablation top and bottom width, ablation depth, and aspect ratio. From the result of the geometry analysis, the minimum measured values of aspect ratio and ablation depth were used as structured electrodes. Laser structuring with pitch distances of 112 μm, 224 μm, and 448 μm was applied. Secondly, the wettability of the electrodes was measured mainly by total wetting time and electrolyte spreading area. This study demonstrates that the laser-based structuring of the electrode increases the electrochemically active surface area of the electrode. The electrode structured with 112 μm pitch distance exhibited the fastest wetting at a time of 13.5 s. However, the unstructured electrode exhibited full wetting at a time of 84 s.

## 1. Introduction

The energy demand of daily human activities is growing fast, causing climate change as a result of greenhouse gas emissions. The automotive industry is labeled the primary contributor to this climate change [1]. Automotive battery technology is essential for the production of efficient, sustainable, safe, stable, and low emission hybrid and electric vehicles to overcome the above-mentioned challenges [2]. In contrast to lead-acid batteries and nickel-cadmium batteries, lithium-ion batteries have high energy and power density, a lighter weight, are nontoxic, and have a long cycle life [2,3,4,5,6].

The main components of a lithium-ion battery are the anode, cathode, separator, and electrolyte. The anode is the negative electrode, which usually uses graphite as an active electrode material. The active electrode material is coated on a copper current collector foil. The cathode, meanwhile, is made of lithium metal oxide that coats the aluminum current collector. During the battery charging process, lithium ions and electrons flow from the cathode to the anode through an external circuit and separator, and vice-versa during discharging [7,8,9]. As mentioned above, the active material of the battery are the main elements that determine the performance of the battery. LiFePO_4_ (lithium iron phosphate) is one of the most widely used cathode materials in energy storage. It offers excellent electrochemical performance because of its low cost and thermal stability, as well as the fact that it is environmentally safe compared to other cathode materials [10].

During the past few years, technologies that have been used for cutting, welding, annealing, structuring, and surface processing of lithium-ion batteries have been replaced with a new and simple method using a laser, as the laser is a non-contact process and can perform precision processes. 2D electrode designs exhibit long ion transport distances, power losses, and interelectrode ohmic resistance, which results in the restricted performance of the battery by minimizing energy and power densities. To overcome these disadvantages, researchers have come up with the idea of the 3D electrode. 3D electrode architectures are obtained by removing the active material partially and increasing the areal energy capacity to overcome the above-mentioned limitations and mechanical degradation during battery operation [9,11,12]. A battery’s performance is mainly determined by its electrode performance, and laser structuring of an electrode is mainly aimed at improving this.

Wettability is one of the main properties that greatly affects the electrode’s performance, as insufficient and non-uniform wettability of an electrode results in poor capacity, production failure, reduced lifetime, and degradation [9,13,14,15]. In the past, numerous studies have attempted to investigate electrode surface characteristics and their effect on wettability. The first study analyzed the effect of electrode calendering and found that calendering of the electrode changes the pore structure, thereby improving the wetting behavior [11,16]. The wetting behavior of laser-induced electrode surface structures such as holes, cone, grid, and line structures were studied, and results indicated that surface modification has a huge impact on improving wettability [12]. Other studies looked at the effect of electrolyte solution on the wettability of electrodes, and found that the wetting rate mainly depends on the viscosity and surface tension of the electrolyte solution [17]. However, most studies have focused on the effect of electrolytes with different compositions on wettability, and there is a lack of study on the effect of laser processed electrode surface on wettability. Hence, this study aims to investigate the effect of the electrode surface microstructure. In addition, a wettability test is conducted to compare the wetting behavior of micro-structured electrodes.

In the present experiment, the effect of laser power on the structured electrode was observed in terms of ablation width, ablation depth, and aspect ratio. The structured electrode microstructure was analyzed using SEM. In addition, the wettability of unstructured and structured electrodes was analyzed in terms of wetting time and spread area. This paper is organized as follows. First, an experimental setup, material information, laser source, and laser parameters are described. Second, the surface morphology, ablation width, ablation depth, and aspect ratio are discussed. Third, the wettability of the electrodes is explained and discussed. Finally, the concluding remarks of this study are summarized.

## 2. Experimental Setup

### 2.1. Material

For this study, a one-side coated LiFePO_4_ electrode with aluminum as a current collector was used. The slurry was prepared by mixing active material LiFePO_4_, super P as a conducting agent, and polyvinylidene fluoride as a binder with a mass fraction of 8:1:1, respectively. Active material with a thickness of 70 μm was coated on 20 μm of aluminum foil. After calendering, the electrode total thickness was reduced to 76 μm. At the same time, the active electrode material thickness was also reduced to 56 μm. A LiPF_6_ liquid electrolyte composed of a solvent mixture of ethylene carbonate (EC) and ethyl methyl carbonate (EMC) with a volume ratio of 3:7 was used for the wettability test. The measurements were carried out as static experiments under ambient air using a drop volume of 0.01 mL [18].

### 2.2. Laser Source

**Figure 1:**
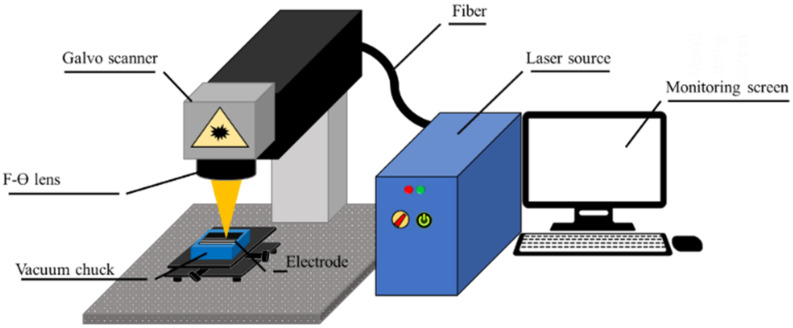
Set up of Ytterbium nanosecond Pulsed Fiber Laser used for laser structuring.

A Ytterbium nanosecond pulsed fiber laser (YLPM-1-4 × 200- 20-20, IPG, NY, New York, USA), shown in Figure 1, was used as a laser source for this experiment. It features a maximum average laser power of 20 W, an emission wavelength of 1064 nm, and a maximum pulse duration of 200 ns. The laser source also had a beam quality factor (M^2^) of 1.5, a collimated beam diameter of 7.2 mm, and a spot diameter of 30 μm.

### 2.3. Experiment Procedure

The LiFePO_4_ electrode was structured with a two-pass method using a laser power of 1–4.6 W (at 0.4 W intervals). The electrode structure with the minimum groove size was chosen for further laser structuring with three different pitch distances of 112 μm, 224 μm, and 448 μm. To evaluate the wettability of structured and unstructured electrodes, electrolyte spread area and total wetting time were analyzed. The effect of surface microstructure on the wetting behavior of both a structured and an unstructured electrode was investigated. The experiment parameters employed for this study are indicated in Table 1.

## 3. Result and Discussion

### 3.1. Morphology

**Figure 2:**
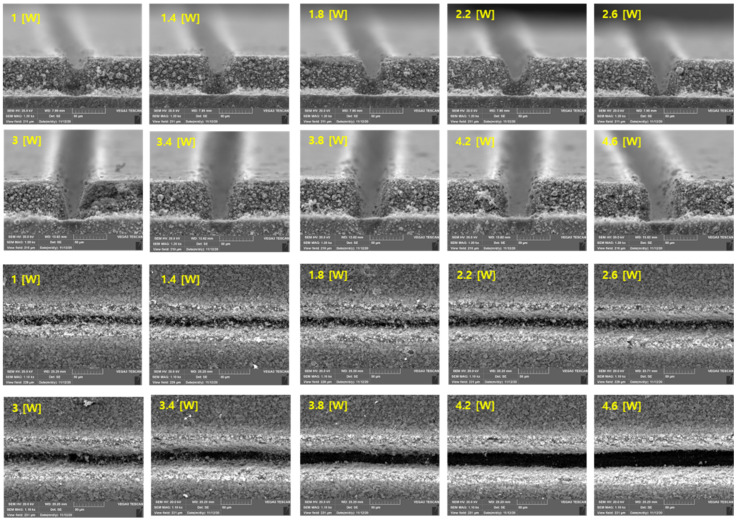
SEM image of the electrode after the laser structuring with two-pass.

A scanning Electron Microscope (SEM) was employed to observe the top and cross-section macrostructure of the electrode structures as shown in Figure 2. From both top and cross-section views, the formation of a clear groove was observed from the minimum laser power of 1 W. The spherical solidified molten active electrode material of approximately 5–8 μm in size was observed from the laser power of 1 W. The size of these melt formations increases as the laser power increases. Under higher laser powers, observation reveals an increase in molten active electrode materials extant on the top surface.

### 3.2. Ablation Width, Ablation Depth, and Aspect Ratio

**Figure 3:**
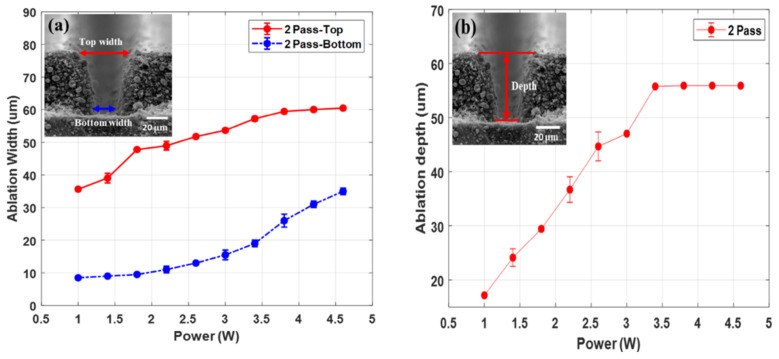

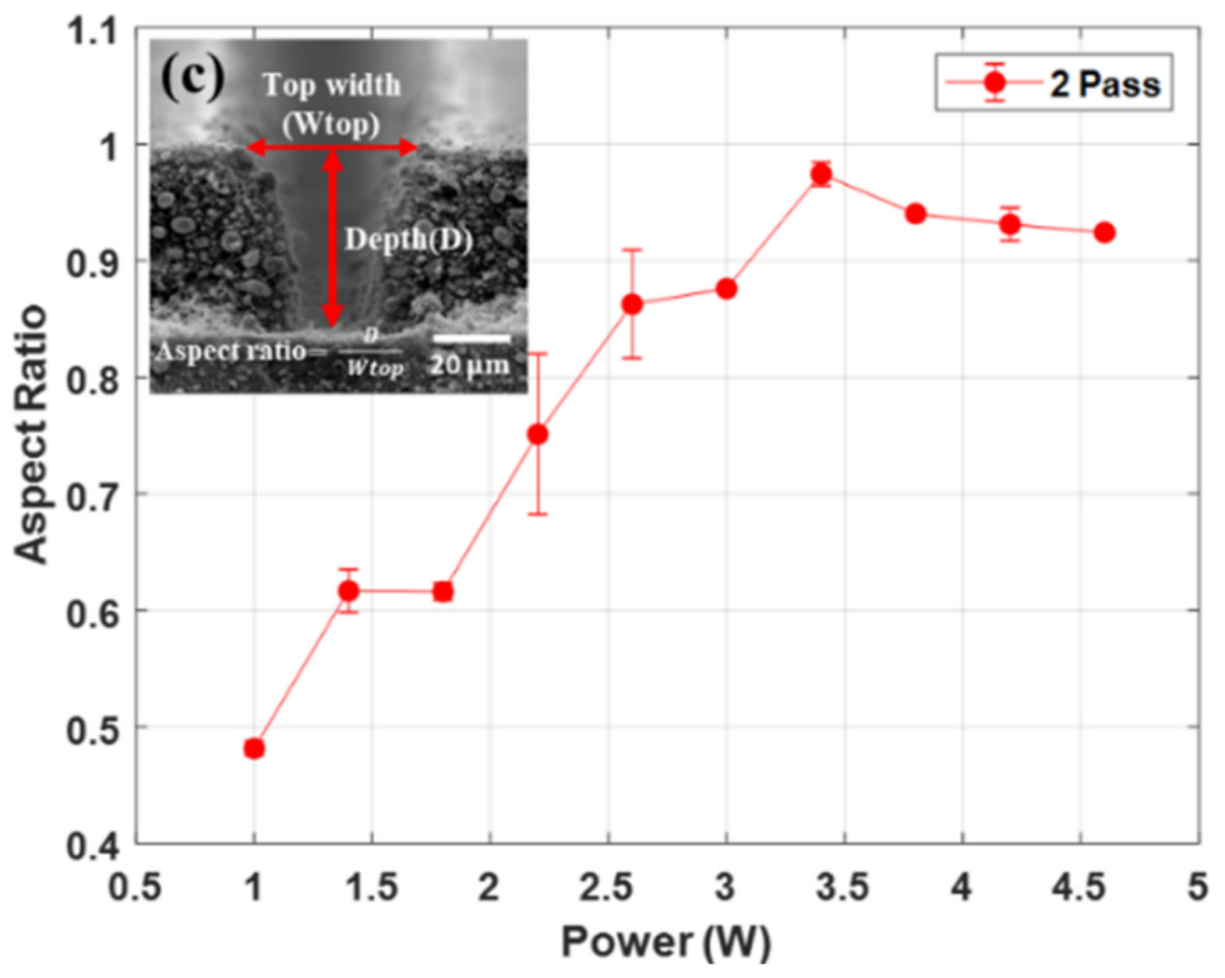
(**a**) Ablation width, (**b**) ablation depth, and (**c**) aspect ratio.

The ablation top and bottom widths after being subjected to all laser powers in are shown in Figure 3a. When the laser power increases, both the ablation top and bottom width increase. The measured ablation top width ranges from 35 μm to 60 μm when using laser powers from 1 W to 4.6 W. Meanwhile, the ablation bottom width varies from 8 μm to 35 μm over the same power range. From the laser power of 1 W to 3.8 W, the ablation top width increases rapidly. On the other hand, the bottom width increases significantly at the laser power ranging from 3 W to 4.6 W.

Figure 3b,c show the ablation depth and aspect ratio of the laser structured electrode. An ablation depth of 17 μm is formed at the laser power of 1 W. The ablation depth increases as the laser power increases. The maximum ablation depth is obtained at the laser power of 3.4 W, at which point the aluminum foil gets exposed. The measured maximum ablation depth is 56 μm. The ablation depth remains stable for the laser powers from 3.4 W to 4.6 W because the active electrode material is completely removed.

The reason ablation depth does not change after the active electrode material is fully ablated indicates that the fluence of the laser beam cannot exceed the ablation threshold of the aluminum foil [17]. The laser powers from 3.4 W to 4.6 W contribute to the increase of the ablation top and bottom width, but do not affect the ablation depth. The ablation depth has reached its maximum possible value at the laser power of 3.4 W. The aspect ratio calculated using the proportion of ablation depth to ablation top width is shown in Figure 3c. The aspect ratio is a very important parameter. The maximum aspect ratio results in creating an increased active electrode material surface area for better electrochemical reaction and enhanced energy density of the battery [18]. The maximum average aspect ratio was found to be 0.974, and was observed when the laser power of 3.4 W was used. The maximum aspect ratio is obtained when the active electrode material is completely removed. Moreover, the aspect ratio decreases as the laser power increase from 3.4 W. The reason is that the ablation width keeps increasing while the ablation depth remains unchanged. As the result, the aspect ratio declines. To clearly understand the wettability phenomenon between structured and unstructured from the start, the groove with the smallest depth and aspect ratio measurements was chosen. The groove formed at the laser power of 1 W is structured again for further wettability tests. It was laser structured using three different pitch distances of 112 μm, 224 μm, and 448 μm.

### 3.3. Wettability

**Figure 4:**
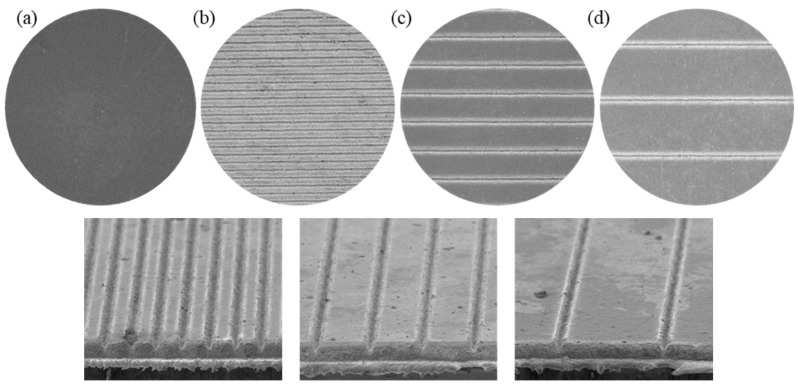
(**a**) Unstructured, (**b**) pitch distance of 112 μm, (**c**) pitch distance of 224 μm, and (**d**) pitch distance of 448 μm.

Wetting property is dependent on drop spreading, electrolyte imbibition, and wicking in the surface [16]. The electrolyte diffusion rate of a battery cell contributes significantly to its capacity, safety, and life cycle. Moreover, the wetted volume of drop and wetting time are the main factors of electrolyte diffusion [16]. The structures formed on the surfaces of the electrodes result in a significant increase in the electrode surface area. According to Wenzel, the wettability of the structured electrodes can be improved by modifying the electrode’s surface (i.e., increasing surface roughness) [16,17,19,20]. In this study, electrodes wettability was compared using the total wetting time and spread area at specific intervals. The unstructured electrode and structured electrodes at a laser power of 1 W with different pitch distances are shown in Figure 4.

Wettability is evaluated in terms of the wetting time and spreading area. The total wetting time is the time required for the electrolyte to fully penetrate the electrode. The spreading area of the electrolyte is the area covered by the liquid electrolyte while diffusing over the electrode surface before fully penetrating the electrode. Additionally, the spread area measurement starts from the initial drop to the spread area where a faintly visible contact angle between the electrode surface and electrolyte drop can be obtained at different times, t.

**Figure 5:**
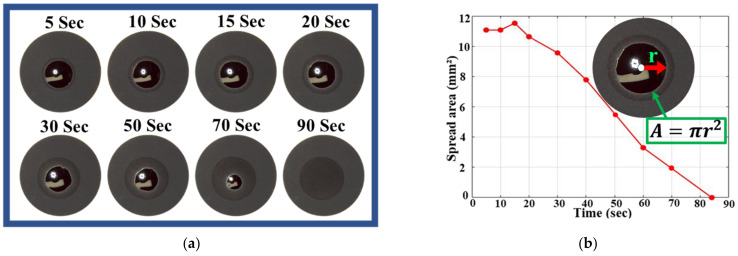
(**a**) Images of an unstructured electrode at different points in time. (**b**) Electrolyte spread over the unstructured electrode with time.

**Figure 6:**
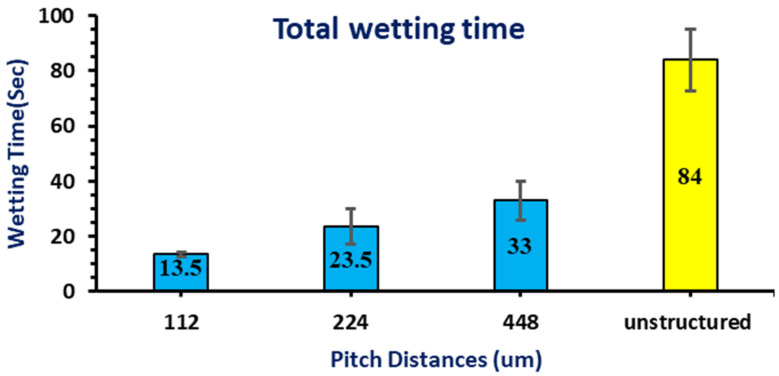
Total wetting time of all electrodes.

Figure 5 shows the electrolyte spread area at different wetting times and expresses the quantitative electrolyte covered area. Additional pictures for the unstructured electrode at 30, 50, 70, and 90 s were also taken. The total time required for the electrolyte to fully penetrate the unstructured electrode was 84 s. In Figure 6, the total wetting time for the electrode structured with 112 μm pitch was 13.5 s, the electrode structured with 224 μm was 23.5 s, and the electrode structured with 448 μm was 33 s. The wetting time difference between the structured electrodes is approximately 10 s. However, the unstructured electrode took a long time compared to the structured electrodes.

Taken together, these results indicate that the wetting time of structured electrodes increases when the pitch distance between grooves increases. Electrodes with small pitch distances have less wetting time than those with bigger pitch distances. This is due to an increased active surface area caused by smaller pitch distances.

**Figure 7:**
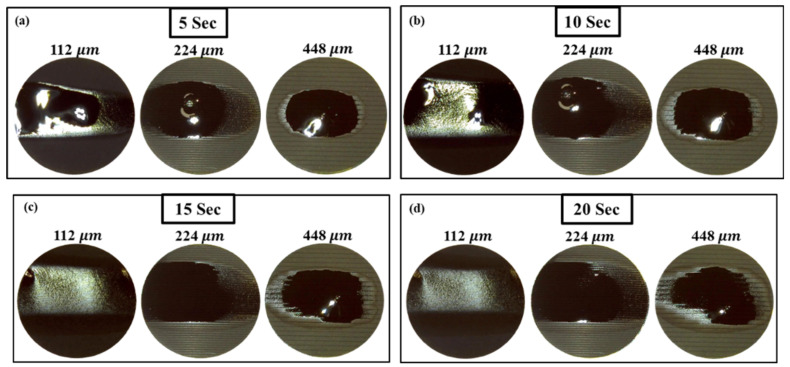
Images of the laser structured electrode with pitch distances of 112 μm, 224 μm, and 448 μm, respectively at the time of (**a**) 5 s, (**b**) 10 s, (**c**) 15 s, and (**d**) 20 s.

Moreover, the wettability is also evaluated by analyzing the wetted surface area after 5, 10, 15, and 20 s, respectively. The unstructured electrode showed different wettability phenomena, unlike the structured electrode. The electrolyte droplet is stable on the surface and does not spread. Rather, it wets uniformly in all directions in a circular shape. In the unstructured electrode, the drop is radially symmetrical around the center which is due to the uniform pore distribution of the electrode. In addition, the spread area starts falling with time without showing much difference from the initial drop. The drop spreads over a maximum area of 15 ${\mathrm{mm}}^{2}$ out of the total area of 94 ${\mathrm{mm}}^{2}$. The covered area while spreading can be seen in Figure 7b with time until it becomes fully wet. The images of the structured electrode recorded at 5 s indicate that the spread area of the liquid on the structured electrode with 112 μm pitch distance is larger than the two other structured electrodes. It is also seen that the electrolyte spread area decreases while increasing the pitch distance between grooves.

In comparison with the electrodes with 112 μm and 224 μm pitch distances, the electrode with a 448 μm pitch distance seems to have an elliptical shape spread area and is stable in a fixed zone. After 10 s, more than half of the electrolyte drop has wetted in the electrode with a 112 μm pitch distance structure while the other two are still spreading. After 15 and 20 s, the entire drop placed on the electrode structure with a 112 μm pitch distance is fully spread, and has even started drying while the others are in the process of wetting. The electrolyte spreads along lines parallel to the direction of the line structures. Unlike unstructured electrodes, the spread area of the structured electrodes, in general, is not radially symmetrical because the groove is formed, and the pore distribution is affected by laser structuring. It is seen that the electrolyte spreads and wets quickly on the structured electrode with the smallest pitch distance compared to bigger pitch distances. This implies that the active electrode material surface area increases as the pitch distance gets smaller.

## 4. Conclusions

In this study, LiFePO_4_ electrodes were structured using a nanosecond pulsed fiber laser via two different number passes and three different pitch distances. The wettability of the unstructured electrode and structured electrodes was discussed comparatively. The wettability of the electrodes was mainly measured and analyzed by the electrolyte’s ability to penetrate quickly and its spread area. The key observations of this study can be summarized as follows:

1. The ablation top and bottom widths increase as the laser power increases. Similarly, ablation depth also increases as the laser power increases. The aspect ratio increases as the laser power increases until it starts falling at the laser power of 3.4 W, where the depth reaches its maximum limit while width keeps increasing.
2. The maximum ablation top width was measured to be 60 μm at 4.6 W, and the maximum ablation depth was 56 μm starting from a laser power of 3.4. The minimum measurements were found when using a laser power of 1 W. 35 μm and 17 μm were the minimum measured values of ablation width and depth, respectively. The maximum aspect ratio of 0.974 was obtained using a laser power of 3.4 W.
3. The wettability test results obtained by comparing the unstructured and structured electrodes showed that the surface microstructure influences the wetting performance of the electrode. It was observed that the electrode structured with a small pitch distance exhibits fast wetting and spreading behavior. It has also been noticed that the groove structures play an important role in guiding the electrolyte flow direction. The unstructured electrode shows very slow wetting and a small spread area compared to the structured electrodes.
4. This study demonstrated that the electrolyte drop chooses a differently shaped spread pattern to minimize the energy required for spreading depending on the pattern and geometry of the structured electrodes [18].

Further study may focus on investigating the wettability of laser-induced electrodes and their effect on battery performance using better techniques.

## Figures and Tables

**Table 1:** Experiment parameters.

Laser Parameters	
Laser power(W)	1~4.6 W
Wavelength	1064 nm
Pulse duration	4 ns
Pulse repetition rate	500 kHz
Scanning speed	500 mm/s
Number of passes	2 passes
Pitch distances	112 μm, 224 μm and 448 μm
Working distance	189 mm
